# Ovarian Hyperstimulation Syndrome Caused by Functional Gonadotroph Pituitary Adenoma

**DOI:** 10.1210/jcemcr/luad087

**Published:** 2023-07-14

**Authors:** Ryo Tsukaguchi, Masashi Hasebe, Sachiko Honjo, Akihiro Hamasaki

**Affiliations:** Department of Diabetes and Endocrinology, Medical Research Institute Kitano Hospital, Osaka, 530-8480, Japan; Department of Diabetes and Endocrinology, Medical Research Institute Kitano Hospital, Osaka, 530-8480, Japan; Department of Diabetes and Endocrinology, Medical Research Institute Kitano Hospital, Osaka, 530-8480, Japan; Department of Diabetes and Endocrinology, Medical Research Institute Kitano Hospital, Osaka, 530-8480, Japan

**Keywords:** ovarian hyperstimulation syndrome, functional gonadotroph pituitary adenoma

## Abstract

Functional gonadotroph adenomas (FGAs) are rare, manifesting symptoms like menstrual irregularities or ovarian hyperstimulation syndrome (OHSS). We present a case of OHSS caused by an FGA during the follow-up of a pituitary tumor initially considered nonfunctioning. The patient presented with lower abdominal pain, abdominal swelling, and dyspnea. Magnetic resonance imaging (MRI) revealed 15 cm enlarged ovarian cysts and pleural effusion. Laboratory examination showed an elevated serum estradiol (E2) level (5741.4 pmol/L [1564.0 pg/mL]), suppressed luteinizing hormone, and nonsuppressed follicular-stimulating hormone (FSH). However, no pituitary hormone disorders were observed when a 19 mm pituitary tumor was discovered 11 months prior. Given the absence of human chorionic gonadotropin (hCG) administration, OHSS due to the FGA was suspected. Cabergoline, known for alleviating the severity of OHSS, was administered, but the ovarian cysts continued to enlarge. Subsequently, endoscopic transsphenoidal surgery was performed, and immunohistochemical analysis confirmed the diagnosis of the FSH-producing adenoma. Follow-up MRI scans showed reduced ovarian cysts and successful pituitary tumor resection with a reduced E2 level. This case highlights the importance of considering FGAs when encountering OHSS without hCG administration or following up on pituitary tumors in premenopausal female patients to take appropriate measures for accurate diagnosis and management.

## Introduction

Most immunohistochemically gonadotroph-positive pituitary adenomas are clinically diagnosed as nonfunctioning [1]. Functional gonadotroph adenomas (FGAs) are rare and can present symptoms such as menstrual irregularities or ovarian hyperstimulation syndrome (OHSS) [2]. OHSS is generally a complication of assisted reproductive technology (ART) using human chorionic gonadotropin (hCG), characterized by enlarged ovaries and an acute fluid shift from the intravascular space to the third space [3]; however, there are limited reports of OHSS caused by FGAs [2].

Herein, we describe a patient who developed OHSS due to an FGA during the follow-up of a pituitary tumor initially considered nonfunctioning. Our case highlights the possibility of FGAs in cases of OHSS without hCG administration or during the surveillance of pituitary tumors in premenopausal females.

## Case Presentation

**Figure 1.**
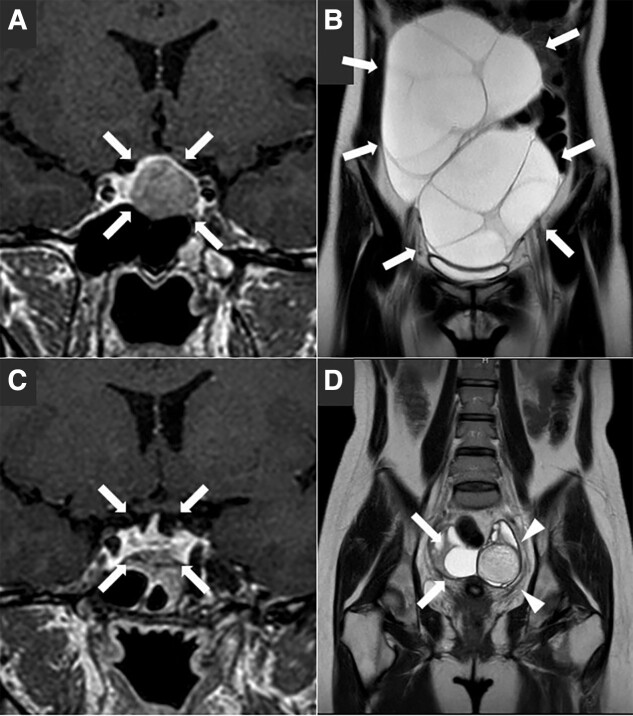
Coronal sections of pituitary or pelvic magnetic resonance imaging. A, A pituitary tumor with a maximum dimension of 19 mm (arrows) before surgery. B, Markedly enlarged multiple ovarian cysts of 15 cm (arrows) at presentation. C, Successful resection of the pituitary tumor (arrows) four months after surgery. D, Noticeably reduced ovarian cysts (arrows) and a 5 cm nodule suspected of a mature cystic teratoma (arrowheads) one month after the pituitary tumor resection.

A 28-year-old woman (height 159.0 cm, weight 52.0 kg, body mass index 20.6 kg/m^2^) presented to our hospital with lower abdominal pain, abdominal swelling, and dyspnea. She was undergoing a follow-up for a previously discovered 19 mm pituitary tumor (Fig. 1A), which had been detected during an examination for a headache 11 months prior. Initially, the tumor was considered nonfunctioning since no apparent pituitary hormone disorders were observed without estradiol (E2) measurements (Table 1). She had a history of bipolar disorder since age 23 and was effectively managed on a medication regimen of lamotrigine 100 mg and brotizolam 0.25 mg at presentation. There was no apparent alteration in the clinical phenotype and no need for dose adjustment of these medications. She had no other medical history, no family history of endocrine disorders, and no prior history of pregnancy or miscarriage. However, she experienced her first menstruation at age 17, and her menstrual cycle was irregular, occurring three or four times per year. Her last menstrual period was 56 days ago at presentation. On physical examination, abdominal swelling and a mass from the lower abdomen to the navel level were observed. She did not exhibit symptoms of hyperandrogenism or mass effects from a pituitary tumor, such as visual disturbances or headaches.

**Table 1.** Laboratory investigations at the initial presentation 11 months prior

		Reference range
Serum luteinizing hormone, mIU/mL	9.04	FP: 1.76-10.24, OP: 2.19-88.33, LP: 1.13-14.22
Serum follicle-stimulating hormone, mIU/mL	6.58	FP: 3.01-14.72, OP: 3.21-16.60, LP: 1.47-8.49
Serum prolactin, ng/mL	25.0	4.91-29.32
Serum growth hormone, ng/mL	1.29	0.13-9.88
Serum insulin-like growth factor-1, ng/mL	180	141-328
Plasma adrenocorticotropin, pg/mL	20.40	7.2-63.3
Serum cortisol, μg/mL	14.6	7.07-19.6
Serum thyrotropin, μIU/mL	3.667	0.61-4.23
Serum free thyroxine, ng/dL	1.26	0.75-1.45

Abbreviations: FP, follicular phase; LP, luteal phase; OP, ovulatory phase.

## Diagnostic Assessment

**Figure 2.**
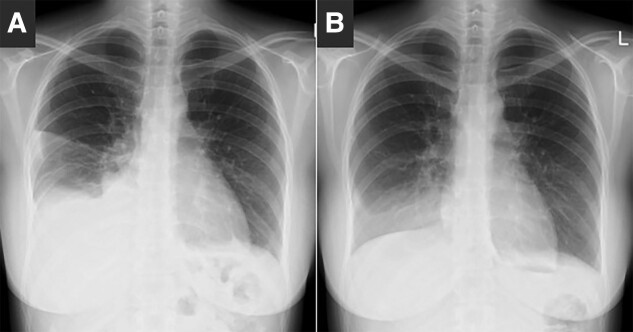
Chest x-ray. A, Bilateral right-dominant pleural effusion at presentation. B, Remarkably improved pleural effusion three days after cabergoline administration.

Noncontrast magnetic resonance imaging (MRI) revealed an approximately unchanged pituitary tumor and markedly enlarged multiple ovarian cysts measuring 15 cm (Fig. 1B). Additionally, a chest x-ray demonstrated bilateral right-dominant pleural effusion (Fig. 2A). Laboratory investigations (Table 2) showed a markedly elevated serum E2 level (5741.4 pmol/L [1564.0 pg/mL]), suppressed luteinizing hormone (LH) level (<0.30 mIU/mL), and nonsuppressed follicular-stimulating hormone (FSH) level (4.86 mIU/mL), consistent with hormonal abnormalities observed in FSH-producing pituitary tumors [2]. In contrast, a serum testosterone level was reduced (<0.10 nmol/L [<0.03 ng/mL]), likely attributed to LH suppression as a consequence of negative feedback from the elevated E2 levels. A serum prolactin level was elevated (1377.6 mIU/L [65.60 ng/mL]), an insulin-like growth factor-1 level was reduced (15.9 nmol/L [121 ng/mL]), and other anterior pituitary hormones were within normal limits. Among tumor markers (see Table 2), only serum carbohydrate antigen 125 level was elevated (208 U/mL), and a plasma D-dimer level was also elevated (4.5 μg/mL), indicating a hypercoagulable state. Based on these findings, we suspected OHSS caused by FSH production from the pituitary tumor, considering the absence of exogenous hCG administration.

**Table 2.** Laboratory investigations at presentation

		Reference range
Serum luteinizing hormone, mIU/mL	<0.30	FP: 1.76-10.24, OP: 2.19-88.33, LP: 1.13-14.22
Serum follicle-stimulating hormone, mIU/mL	4.86	FP: 3.01-14.72, OP: 3.21-16.60, LP: 1.47-8.49
Serum estradiol, pg/mL	1564.0	FP: 28.8-196.8, OP: 36.4-525.9, LP: 44.1-491.9
Serum progesterone, ng/mL	39.9	FP: ≤0.28, OP: ≤5.69, LP: 2.05-24.2
Serum prolactin, ng/mL	65.60	4.91-29.32
Serum growth hormone, ng/mL	1.78	0.13-9.88
Serum insulin-like growth factor-1, ng/mL	121	137-320
Serum testosterone, ng/mL	<0.03	0.11-0.47
Plasma adrenocorticotropin, pg/mL	24.70	7.2–63.3
Serum cortisol, μg/mL	10.1	7.07-19.6
Serum thyrotropin, μIU/mL	1.652	0.61-4.23
Serum free thyroxine, ng/dL	0.83	0.75-1.45
Urine human chorionic gonadotropin	Negative	Negative
Serum carbohydrate antigen 125, U/mL	208	≤35.0
Serum carcinoembryonic antigen, ng/mL	1.6	≤5.0
Serum carbohydrate antigen 19-9, U/mL	<2.0	≤37.0
Serum squamous cell carcinoma antigen, ng/mL	0.6	≤2.5
Serum α-fetoprotein, ng/mL	<2.0	≤10.0

Abbreviations: FP, follicular phase; LP, luteal phase; OP, ovulatory phase.

## Treatment

**Figure 3.**
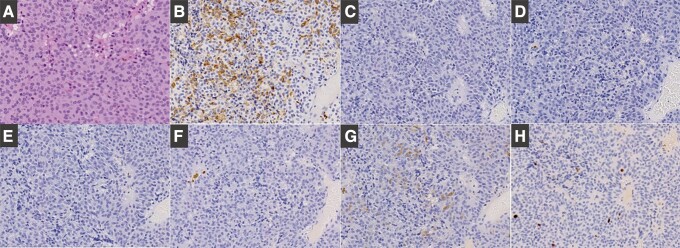
Pituitary tumor pathology including immunohistochemical staining. A, Hematoxylin-eosin staining. B, Positive for follicle-stimulating hormone. C, Negative for luteinizing hormone. D, Negative for prolactin. E, Negative for adrenocorticotropin. F, Negative for growth hormone. G, Negative for thyrotropin. H, The Ki-67/MIB-1 labeling index was determined to be 5%.

In addition to saline, cabergoline, which can alleviate the severity of OHSS [4], was administered. Although the pleural effusion improved (Fig. 2B) and the E2 level reduced to 183.9 pmol/L (50.1 pg/mL), the ovarian cysts continued to enlarge, eventually reaching 25 cm. Subsequently, following the improvement in the pleural effusion and the hypercoagulable state, endoscopic transsphenoidal surgery was performed. Immunohistochemical analysis confirmed positivity for FSH and negativity for other anterior pituitary hormones (Fig. 3). The Ki-67/MIB-1 labeling index was determined to be 5%.

## Outcome and Follow-up

**Figure 4.**
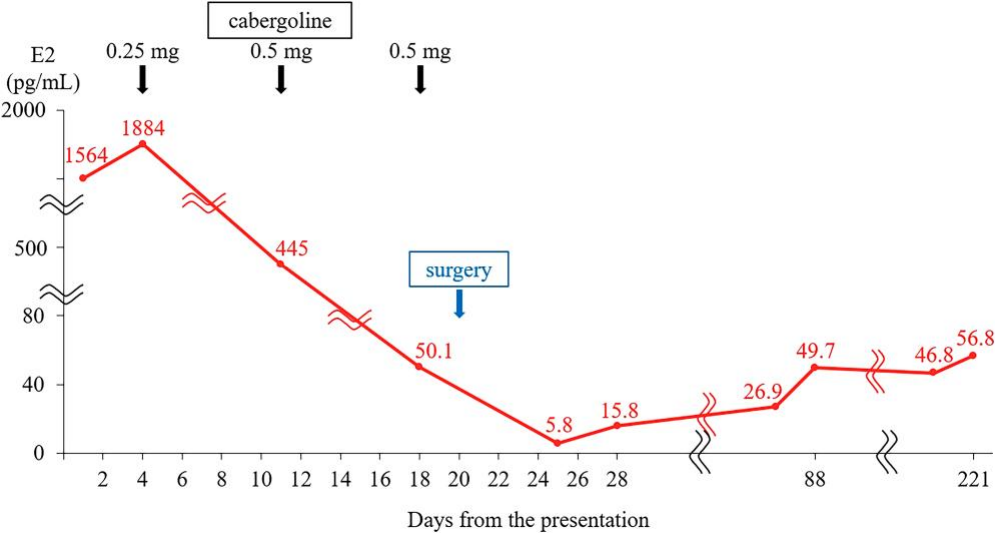
The clinical course of serum estradiol (E2) level.

Within one week after the surgery, the ovarian cysts decreased to 11 cm, and the E2 level reduced to 21.3 pmol/L (5.8 pg/mL), accompanied by an increase in LH level (0.95 mIU/mL) and a decrease in FSH level (1.76 mIU/mL) (Fig. 4). In the postoperative endocrine assessment (Table 3), gonadotropin levels showed no paradoxical response for the thyrotropin-releasing hormone (TRH) test (Table 4) and exhibited a normal response for the LH-releasing hormone test (Table 5). The prolactin level and insulin-like growth factor-1 level normalized, and other anterior pituitary hormones had no apparent disorders. The antimüllerian hormone level was elevated at 132.1 nmol/L (18.5 ng/mL) (reference range, 6.0-88.8 nmol/L [0.84-12.44 ng/mL]).

**Table 3.** Laboratory investigations one week after surgery

		Reference range
Serum luteinizing hormone, mIU/mL	0.95	FP: 1.76-10.24, OP: 2.19-88.33, LP: 1.13-14.22
Serum follicle-stimulating hormone, mIU/mL	1.76	FP: 3.01-14.72, OP: 3.21-16.60, LP: 1.47-8.49
Serum estradiol, pg/mL	5.8	FP: 28.8-196.8, OP: 36.4-525.9, LP: 44.1-491.9
Serum progesterone, ng/mL	0.26	FP: ≤0.28, OP: ≤5.69, LP: 2.05-24.2
Serum prolactin, ng/mL	0.23	4.91-29.32
Serum growth hormone, ng/mL	1.39	0.13-9.88
Serum insulin-like growth factor-1, ng/mL	214	137-320
Serum testosterone, ng/mL	N/A	0.11-0.47
Plasma adrenocorticotropin, pg/mL	29.50	7.2-63.3
Serum cortisol, μg/mL	8.9	7.07-19.6
Serum thyrotropin, μIU/mL	1.384	0.61-4.23
Serum free thyroxine, ng/dL	0.94	0.75-1.45

Abbreviations: FP, follicular phase; LP, luteal phase; N/A, not available; OP, ovulatory phase.

**Table 4.** Results of thyrotropin-releasing hormone (0.2 mg) stimulation test

	Reference range for basal value	0 min	30 min	60 min	90 min	120 min
Serum thyrotropin, μIU/mL	0.61-4.23	1.606	14.071	12.140	9.669	6.957
Serum prolactin, ng/mL	4.91-29.32	0.45	3.51	2.22	1.33	0.87
Serum luteinizing hormone, mIU/mL	FP: 1.76-10.24	3.00	3.62	3.06	3.88	3.41
	OP: 2.19-88.33					
	LP: 1.13-14.22					
Serum follicle-stimulating hormone, mIU/mL	FP: 3.01-14.72	3.34	3.42	3.31	3.39	3.34
	OP: 3.21-16.60					
	LP: 1.47-8.49					

Abbreviations: FP, follicular phase; LP, luteal phase; OP, ovulatory phase.

**Table 5.** Results of gonadotropin-releasing hormone (0.1 mg) stimulation test

	Reference range for basal value	0 min	30 min	60 min	90 min	120 min
Serum luteinizing hormone, mIU/mL	FP: 1.76-10.24	2.77	12.00	12.00	10.30	8.92
	OP: 2.19-88.33					
	LP: 1.13-14.22					
Serum follicle-stimulating hormone, mIU/mL	FP: 3.01-14.72	3.64	6.40	7.08	7.15	7.02
	OP: 3.21-16.60					
	LP: 1.47-8.49					

Abbreviations: FP, follicular phase; LP, luteal phase; OP, ovulatory phase.

Follow-up MRI scans after surgery showed successful pituitary tumor resection (Fig. 1C) and noticeably reduced ovarian cysts with a residual 5 cm nodule suspected of a mature cystic teratoma (Fig. 1D). Furthermore, laboratory investigations conducted over three months after surgery showed a normal E2 level (146.8-183.6 pmol/L [40–50 pg/mL]), LH level (13-14 mIU/mL), and FSH level (7.0-8.0 mIU/mL). The patient's menstrual cycle remained irregular, with intervals of one to two months, shorter than before. Considering the irregular menstrual cycle since the first menstruation and the relatively high LH level after the surgery, there may be an underlying background of polycystic ovary syndrome (PCOS), despite the absence of signs of hyperandrogenism. High antimüllerian hormone level was also potentially attributed to underlying PCOS [5]. However, nine months later, serum testosterone was first measured after the surgery and normalized (0.83 nmol/L [0.24 ng/mL]), not presenting with the high testosterone levels typically associated with PCOS. A summary of the clinical course and treatment is presented in Fig. 4.

## Discussion

In the present case, we characterized OHSS caused by an FGA, presenting with markedly enlarged multiple ovarian cysts and pleural effusion. Administration of cabergoline improved the pleural effusion but did not affect the enlarged ovarian cysts. Subsequently, surgical intervention resulted in a marked reduction of the ovarian cysts. To our knowledge, this is the first reported case of FGAs identified during the follow-up of pituitary tumors initially considered nonfunctioning.

OHSS is commonly known as a complication of ART with hCG administration. It is characterized by the enlargement of ovarian cysts and an acute fluid shift from the intravascular space to the third space. This shift is caused by increased systemic vascular permeability, resulting in decreased circulating blood volume, hemoconcentration, pleural effusion, and ascites [3]. Vascular endothelial growth factor is considered the primary mediator of vascular permeability in OHSS, produced in granulosa cells following hCG administration. Although rare, OHSS can occur spontaneously, particularly in spontaneous pregnancy with hypothyroidism, PCOS, or FSH receptor mutations [6] or as a result of FGAs. We retrospectively postulate that OHSS in the present case resulted from the FSH-producing pituitary adenoma, with the underlying PCOS serving as a potential contributing factor.

Most gonadotropin-producing pituitary adenomas are clinically nonfunctioning, as reported in a case series involving 213 clinically nonfunctioning pituitary adenomas, 64% of which were immunohistochemically gonadotroph adenomas [1]. FGAs are rare and can present with symptoms such as menstrual irregularities, infertility, galactorrhea, or OHSS in premenopausal women, testicular enlargement or hypogonadism in men, as well as isosexual precocious puberty in children [2]. Due to their infrequency, limited case reports are available on FGAs. A systematic review of 65 premenopausal females indicated that FGAs were mainly FSH predominant (92.3%) and macroadenomas (89.2%), with nearly all FSH-predominant FGAs leading to OHSS (98.2%) [7]. The characteristics in the present case were consistent with these reported features. Furthermore, the present case exhibited a markedly elevated E2 level, suppressed LH levels, and normal FSH levels, which aligns with endocrinological findings reported in previous cases of FGAs [2]. The TRH test demonstrated a paradoxical rise in serum FSH or LH levels in a few cases. In the present case, the TRH test was not conducted before the surgery due to concerns about the risk of apoplexy. However, postoperative gonadotropin levels showed no paradoxical response for the TRH test (see Table 4) and exhibited a normal response for the LH-releasing hormone test (see Table 5).

Surgical intervention is considered the primary treatment approach for FGAs, restoring normal gonadotropin secretion and improving associated symptoms such as menstrual irregularities, OHSS, and enlarged ovarian cysts [2]. Indeed, spontaneous pregnancies have been reported in some cases following the surgical removal of FGAs [2, 8]. Conversely, medical treatments involving dopamine agonists, somatostatin analogues, or gonadotropin-releasing hormone agonists/antagonists have shown limited effectiveness in inducing tumor shrinkage [8]. Cabergoline, a dopamine agonist commonly used in OHSS cases induced by ART, has been employed to alleviate vascular permeability by dephosphorylating and inactivating vascular endothelial growth factor receptor 2 [9]. While cabergoline has demonstrated some efficacy in OHSS caused by FGAs, its primary effect is reducing vascular permeability and normalizing serum FSH/E2 levels rather than inducing pituitary tumor shrinkage or reducing ovarian cysts [2, 8]. In a limited number of cases, somatostatin analogues have shown potential effectiveness, except for tumor shrinkage [8, 10]. Nevertheless, gonadotropin-releasing hormone analogues have generally demonstrated limited efficacy and, in some cases, may even exacerbate OHSS [2, 8]. Medical treatments are typically used as adjuncts because of their limited efficacy. In the present case, the administration of cabergoline improved the pleural effusion and normalized FSH/E2 levels, but the ovarian cysts did not shrink, consistent with previously reported cases. Consequently, pituitary surgery was performed, significantly improving the OHSS symptoms. This case underscores and reaffirms the crucial role of surgical intervention as the primary approach for addressing the hormonal imbalance caused by FGAs.

We describe a case of OHSS with an FSH-producing pituitary adenoma. Consistent with previous reports, medical treatment alone proved ineffective in managing the condition, highlighting the pivotal role of pituitary surgery in addressing the underlying cause of OHSS associated with FGAs. The present case demonstrates OHSS caused by an FGA during the follow-up of an initially deemed nonfunctioning pituitary tumor. It is noteworthy that the E2 level was not measured until the presentation with OHSS, and therefore its elevation at that time cannot be ruled out. Clinicians should consider FGAs when encountering OHSS without hCG administration or during the follow-up of pituitary tumors in premenopausal female patients for accurate diagnosis and management.

## Learning Points

- Gonadotropin-producing pituitary adenomas can cause OHSS in premenopausal females.
- OHSS caused by FGAs is characterized by the enlargement of ovarian cysts and a rapid fluid shift from the intravascular space to the third space, typically accompanied by an elevated serum E2 level, suppressed LH level, and nonsuppressed FSH level.
- Surgical intervention is the primary treatment modality for FGAs, restoring normal gonadotropin secretion and improving associated symptoms.
- Medical treatments are typically used as complementary measures due to limited evidence of effectiveness. Among them, cabergoline can alleviate OHSS by reducing vascular permeability and normalizing FSH/E2 levels, but it does not lead to pituitary tumor shrinkage or reduction of ovarian cysts.
- Clinicians should consider the possibility of FGAs when encountering OHSS without hCG administration or when monitoring premenopausal females with pituitary tumors.

## Contributors

All authors made individual contributions to authorship. All authors were involved in the diagnosis and management of this patient. R.T. and M.H. were involved in writing the first draft and submission. S.H and A.H critically revised the manuscript with important intellectual content. All authors read and approved the final draft.

## Data Availability

Data sharing is not applicable to this article as no data sets were generated or analyzed during the current study.

## Abbreviations

ART: assisted reproductive technology
E2: estradiol
FGA: functional gonadotroph adenoma
FP: follicular phase
FSH: follicular-stimulating hormone
hCG: human chorionic gonadotropin
LH: luteinizing hormone
LP: luteal phase
MRI: magnetic resonance imaging
OHSS: ovarian hyperstimulation syndrome
OP: ovulatory phase
PCOS: polycystic ovary syndrome
TRH: thyrotropin-releasing hormone
